# The Effect of Human Activities and Their Associated Noise on Ungulate Behavior

**DOI:** 10.1371/journal.pone.0040505

**Published:** 2012-07-10

**Authors:** Casey L. Brown, Amanda R. Hardy, Jesse R. Barber, Kurt M. Fristrup, Kevin R. Crooks, Lisa M. Angeloni

**Affiliations:** 1 Graduate Degree Program in Ecology, Colorado State University, Fort Collins, Colorado, United States of America; 2 Department of Biological Sciences, Boise State University, Boise, Idaho, United States of America; 3 Natural Sounds and Night Skies Division, U.S. National Park Service, Fort Collins, Colorado, United States of America; 4 Department of Fish, Wildlife, and Conservation Biology, Colorado State University, Fort Collins, Colorado, United States of America; 5 Department of Biology, Colorado State University, Fort Collins, Colorado, United States of America; Australian Wildlife Conservancy, Australia

## Abstract

**Background:**

The effect of anthropogenic noise on terrestrial wildlife is a relatively new area of study with broad ranging management implications. Noise has been identified as a disturbance that has the potential to induce behavioral responses in animals similar to those associated with predation risk. This study investigated potential impacts of a variety of human activities and their associated noise on the behavior of elk (*Cervus elaphus*) and pronghorn (*Antilocapra americana*) along a transportation corridor in Grand Teton National Park.

**Methodology/Principal Findings:**

We conducted roadside scan surveys and focal observations of ungulate behavior while concurrently recording human activity and anthropogenic noise. Although we expected ungulates to be more responsive with greater human activity and noise, as predicted by the risk disturbance hypothesis, they were actually less responsive (less likely to perform vigilant, flight, traveling and defensive behaviors) with increasing levels of vehicle traffic, the human activity most closely associated with noise. Noise levels themselves had relatively little effect on ungulate behavior, although there was a weak negative relationship between noise and responsiveness in our scan samples. In contrast, ungulates did increase their responsiveness with other forms of anthropogenic disturbance; they reacted to the presence of pedestrians (in our scan samples) and to passing motorcycles (in our focal observations).

**Conclusions:**

These findings suggest that ungulates did not consistently associate noise and human activity with an increase in predation risk or that they could not afford to maintain responsiveness to the most frequent human stimuli. Although reduced responsiveness to certain disturbances may allow for greater investment in fitness-enhancing activities, it may also decrease detections of predators and other environmental cues and increase conflict with humans.

## Introduction

Anthropogenic noise can impact animals in ways that are only beginning to be explored [1]. Noise is pervasive in both developed and natural areas [2], [3] and can be deleterious to an animal's physiology and behavior. If chronic, it may affect an animal's auditory system [4], increase cardiac and stress levels [5], [6], and impair communication [7]–[11]. Noise can also alter pairing and reproduction [9], [12], age structuring [9], and density and occupancy patterns [13]–[15].

Noise has also been identified as a disturbance that could induce behavioral responses similar to those associated with predation risk [16]. The risk-disturbance hypothesis predicts that animals exposed to anthropogenic disturbance, such as noise, will exhibit antipredator behavior that takes time and energy away from fitness-enhancing activities [16]. Indeed, prior studies have documented behavioral responses, such as vigilance, avoidance, and flight, to anthropogenic noise for a variety of taxa [5], [17]–[20]. An increase in vigilance may be costly if it results in a decrease in maintenance activities such as foraging [21], [22], and displacement or flight may expend valuable amounts of energy [23]–[25]. Thus, noise can affect habitat selection, foraging patterns, and overall energy budgets [26], [27], with potential population-level effects. However, noise may not have lasting negative effects if animals habituate to the disturbance, that is exhibit reduced responsiveness over time after repeated exposure without consequence [28]; e.g., [5], [29]–[31]. In some cases animals may even be attracted to and benefit from noisy disturbed areas, for example if they provide shelter from predators [32]–[35].

Large mammals, such as ungulates, may be particularly sensitive to anthropogenic disturbance [36], [37], including human activities associated with recreation, transportation, ecotourism and the noise they produce [33], [38]–[40]. Recreational activities such as snowmobiling, skiing, biking and hiking can alter the behavior of ungulates [24], [41]–[48]. Roadways can also induce a range of behavioral responses in ungulates, which in some cases seem attracted to or unaffected by road activity [32], [41] but more commonly exhibit risk-avoidance behavior in response to roads [25], [33], [39], [40], [49]–[53]. Although the degree to which animals are responding to visual or acoustic disturbances generated by these recreational and transportation activities remains largely unexplored, there is some evidence for the independent effect of noise, reviewed in [1], [2], [7]; but see [54].

The goal of this research was to quantify the behavioral response of ungulates to a variety of human activities and their associated noise along the primary travel corridor in Grand Teton National Park, USA. We evaluated the effect of human activities and concurrent sound properties on ungulate behavior along this corridor. If, according to the risk disturbance hypothesis [16], activities of park visitors represent a form of predation risk to ungulates, then we predicted ungulates would display heightened responsive behavior with increasing levels of anthropogenic stimuli, including both noise and human activity. Alternatively, the behavior of ungulates along the travel corridor could be unaffected by the level of noise and human activity if they have habituated to human disturbance over time or if sensitive individuals have been previously displaced from this location [55].

## Methods

### Study area

**Figure 1:**
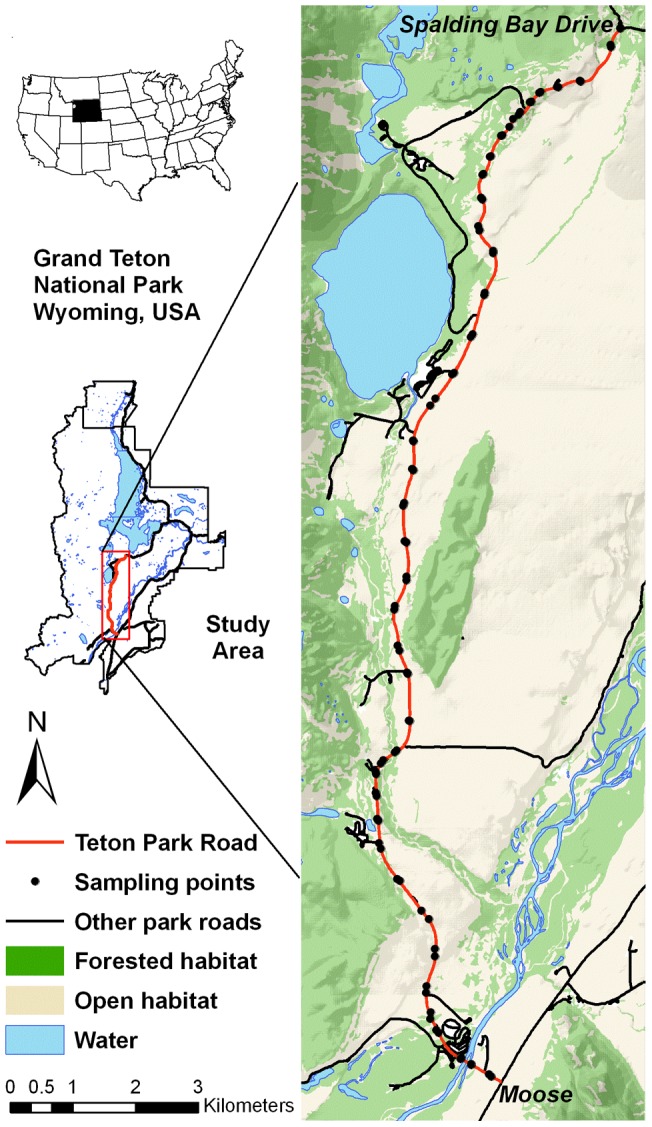
Map of study area.

We conducted the study in summer 2008 along 22 km of Teton Park Road in Grand Teton National Park in northwestern Wyoming, USA (43–50′00″ N, 110–42′03″ W; Figure 1). Teton Park Road is located at the eastern base of the Teton Range and traverses the valley floor from north to south through a predominantly open sage-brush community where large ungulates congregate and visitors often stop to view wildlife. The study area included a stretch of Teton Park Road from its junction with Spalding Bay Drive to its junction with the town of Moose (Figure 1). Our research focused on the two ungulate species most prevalent along the road, elk (*Cervus elaphus*) and pronghorn (*Antilocapra americana*). Large numbers of elk (∼2,500–4,500 [56]) and pronghorn (∼200 [57]) spend the summer in Grand Teton National Park with the potential to move into and out of our study area. The behavior of both species may be influenced by predation risk in this system given the presence of carnivores within the park, including grizzly bear (*Ursus arctos*), black bear (*Ursus americanus*), gray wolf (*Canis lupus*), and mountain lion (*Puma concolor*), although these predators were only rarely observed in our study area. These ungulates also have the potential to experience hunting by humans, particularly when they venture outside our study area during the fall archery and rifle hunting seasons.

### Behavioral Observations

#### Scan sampling

We recorded the behavior of individuals in ungulate herds through scan sampling at 42 points along Teton Park Road (Figure 1) from 14 June 2008 to 18 October 2008. We selected sampling points every 160 to 650 meters to standardize search efforts over space and time and to maximize visible area from the road in an attempt to include the entire viewshed along this stretch of Teton Park Road. Scan sampling occurred during both daytime and crepuscular hours, with staggered starting times to balance sampling effort across periods, allowing at least twelve hours between surveys.

To conduct scan sampling, we drove along Teton Park Road starting at either the northern or southern end of the study area and stopped at each sampling point to scan for ungulate herds with binoculars and a spotting scope. A herd was defined as ≥1 animal present, and a distance of 100 meters was used to delineate different herds, following Childress and Lung [21] who described this as the maximum distance at which elk respond to conspecific vocalizations. Once a herd was sighted, we noted the time of day and counted the number of individuals in the herd. We visually estimated whether the herd was clustered, with most individuals within 25 meters of a nearest neighbor, or dispersed, with most individuals greater than 25 meters from a nearest neighbor; we selected this threshold because it was relatively easy to detect visually and it divided our herds roughly evenly into clustered and dispersed categories. We used laser rangefinders to measure the distance to the center of the herd from the road (our vehicle) and the distance to closest vegetation cover, categorized as near or far to cover (using 100 m as the threshold, a distance across which elk vigilance patterns are known to change [58]).

Once the initial herd data were collected, we recorded behavior only if the herd was within 500 meters of the sampling point to ensure accuracy of behavioral observations. One observer scanned the herd from left to right recording the behavioral category of each individual, following [21], [47]: feeding, grooming (licking or scratching), bedded, mating (sparring or bugling), traveling (walking), fleeing (running), scanning (standing with head above shoulder level), vigilant (displaying alarm or acute attention toward stimuli), and defensive (kicking, biting, charging). Scan surveys lasted approximately 1 minute. It is important to note that ungulates were not tagged or individually identified in our study area; thus, although we can be confident that we sampled unique individuals within each sampling bout as we moved along Teton Park Road, we cannot rule out the possibility that we observed the same individuals on multiple occasions across our scan and focal (described below) sampling bouts.

While ungulate behavioral data were collected, a second observer simultaneously conducted a scan sample to count different kinds of human activity within 200 meters of the sampling point. Ungulates have been shown to be sensitive to the approach speed and direction of anthropogenic stimuli [36]; therefore we categorized vehicles as moving versus stopped. Ungulates can also be particularly responsive to the human form [36]; therefore we also recorded the number of pedestrians along the road. Human activities recorded during scan samples included the number of automobiles (autos) passing, the number of autos stopped (including our own vehicle), and the number of pedestrians at each sampling point. Observers strove to remain in the vehicle to reduce potential observer effects, but on rare occasions when it was necessary to exit the vehicle during a scan observation (e.g., to see a herd that was partially obscured from view), we recorded the observer as a pedestrian to account for our presence and potential influence. We also recorded whether motorcycles, trucks (including recreational vehicles and large commercial and construction vehicles), and bicycles were passing but rarely recorded these activities during our scan samples. Consequently, we did not analyze these three activities separately, but rather grouped passing motorcycles, trucks, and autos into an additional category (total vehicles passing) and omitted passing bicycles from the analyses.

Concurrent with the ungulate and human behavioral observations, we used a portable recording device to sample noise. The recorder (iAudio 7, Cowan America, Irvine, California) was attached to PA3 microphones and a horn lens. The device was mounted on our research vehicle approximately 1.5 meters off the ground and microphones were spaced 2 meters apart pointing in opposite directions. The consistently close proximity of the recorder to the road allowed us to effectively record motorized vehicles, road noise, bicycles, and pedestrians (i.e., human voices). We used a sampling rate of 64 bits per second and recordings were saved as uncalibrated WMA files that could be analyzed for relative metrics of sound. We produced waveforms using SWITCH sound file converter (NCH Software, Canberra, Australia) and spectrograms using RAVEN PRO 1.4 (Cornell University, Ithaca, New York) to quantify relative sound metrics. As the perception of loudness depends on both the amplitude and frequency of sound waves, we measured average power, or the mean relative amplitude over the entire observation, and peak frequency, or the frequency at which the maximum power occurred.

#### Focal Animal Sampling

In addition to scan sampling, we conducted extended behavioral observations of individual focal animals. We initiated focal animal sampling opportunistically, between scan sampling events, as well as systematically, during scheduled daytime and crepuscular focal animal sessions. Observers drove the length of the study area searching for ungulate herds. When a herd was sighted within 500 meters of the road, we recorded its dispersion and location. We randomly selected a focal animal within a herd by counting individuals in the herd from left to right until reaching a chosen random number, and we recorded its sex classification (adult male, adult female or adult female with calf, if a female was in close proximity to or seen tending to a calf). The focal animal observer continuously recorded the behavioral state (same categories as described above) and the timing of any changes in behavioral state for up to 50 minutes or until the focal animal bedded or moved out of view. We excluded focal animal samples with a duration less than 3 minutes (following Childress and Lung [21]) resulting in an average sample duration of 14.6 minutes (SE = 0.8, n = 113).

As with scan samples, we continuously recorded sound for the duration of the focal sample to measure average power and peak frequency. Simultaneously, a second observer alternated between conducting scan samples of behavior for all individuals within the herd and conducting scan samples to count human activities in the vicinity (within 200 meters of the observers). The alternating herd and human activity scans continued throughout the duration of the focal animal sample, with repeated intervals of approximately 45 seconds to 3 minutes; the duration and frequency of scan samples were dependent on herd size and amount of human activity in the vicinity. The herd behavioral scans were conducted for a concurrent study (Hardy, unpublished data); we use only the human activity data here. Anthropogenic activities recorded during focal samples included the number of autos, motorcycles, trucks, and bicycles passing; the number of autos stopped; and the number of pedestrians present.

### Data Analysis

#### Scan sampling

We developed a candidate set of nonlinear mixed models with a binomial distribution (Proc NLMixed, SAS 9.1) to evaluate if and how acoustic variables and human activities predicted the probability that each individual within a herd was responding or not responding, expressed as a binary, categorical variable. Individuals were classified as ‘responding’ if they were vigilant, if they displayed defensive behavior, or if they were fleeing or traveling [47], [59]. Although animals may travel for a variety of reasons, human activity has been observed to provoke movement in general [24], [60] and walking in particular [41], [61]–[63] in a variety of ungulates, including elk within this Greater Yellowstone Ecosystem [47], [64].

Our candidate models included all combinations of five acoustic and human activity predictor variables (average power, peak frequency, total vehicles passing, autos stopped, and pedestrians present). Each model additionally included all of the following covariates that have been shown to influence responsive behavior in ungulates [36], [39], [58], [65], [66]: distance to road, distance to cover, dispersion (clustered versus dispersed), herd size, species (pronghorn or elk), Julian date, and time of day (crepuscular: ≤1 hour after dawn or prior to dusk, or daytime: >1 hour after dawn or prior to dusk, as determined by regional sunrise and sunset tables). We also included the herd ID (a number from 1 to 161 assigned to each scan sample) as a random effect in each model to avoid statistical issues related to pseudoreplication, since an individual's behavior within a scan sample may be correlated with the behavior of the other animals scanned within the same herd. Our candidate model set included an intercept-only model, a covariate model, and models with all subsets of acoustic and human activity predictors in addition to the covariates.

AIC_c_ (Akaike Information Criterion adjusted for small sample size) [67] based on likelihood values were calculated for each model of ungulate herd responsiveness. We reported model weights (*w_i_*) and AIC_c_ differences (Δ), measuring the information loss between models given the data, to compare model ranking. Because our model set was balanced by including all combinations of acoustic and human activity variables, we were able to calculate relative variable importance weights (sum of model weights for all models containing that specific variable) to determine which of these variables were the strongest predictors of ungulate responsiveness [67], [68]. For each predictor, we also calculated model-averaged parameter estimates and their associated 95% confidence intervals to account for model selection uncertainty and to provide unconditional estimates not dependent on a single model [67]. However, because model-averaging might not reliably assess the effect of a single predictor variable [69], [70], we also reported parameter estimates for the predictors in the top model, which necessarily provide conditional estimates, and we calculated estimates from the relationship between each sole predictor and responsiveness, which produce estimates that are not conditional on other predictors.

#### Focal animal sampling

We used linear regressions (Proc Genmod, SAS 9.1) to evaluate the relationship between behavioral budgets of individual animals in the focal observations and acoustic and human activity. For these analyses, the sampling unit was the focal animal and our response variable was the proportion of time spent responding (i.e., vigilant, defensive, fleeing, traveling). Proportionate data was square root arcsine transformed to normalize variance prior to analyses. We calculated overall rates for human activity variables, averaged across all human scans that occurred during a focal observation (i.e., mean number of activities per scan), to adjust for variation in the number of human activity scans conducted while observing focal animals.

To predict focal animal responsiveness, we created candidate models with all combinations of acoustic and human activity predictors (in addition to an intercept-only model and a model with just the covariates), using similar variables as for the scan samples. However, we separated the total passing vehicles into passing autos and motorcycles, and we also included passing bicycles as a distinct predictor, because they were recorded in sufficient frequency in our focal samples due to their longer duration; this resulted in a total of seven acoustic and human activity predictors. All candidate models included the same covariates as in the scan samples, including distance to road, distance to cover, dispersion, herd size, species, Julian date, and time of day. Past studies suggest the sex of an individual may also affect responsiveness [71], [72]; thus we additionally included the focal animal's sex classification. As with the scan samples, we reported AIC_c_ values, model weights, and parameter estimates and confidence intervals from the top model, from model averaging, and from a model where each variable was the sole predictor; variable importance weights were also calculated to determine which acoustic and human activity variables were the strongest predictors of ungulate responsiveness.

## Results

### Scan Samples

Across 161 scan samples, we observed a total of 334 autos stopped, 265 total vehicles passing (including 245 autos, 11 trucks, 9 motorcycles), 135 pedestrians, and 4 bicycles passing. Our uncalibrated measures of average power during scan samples ranged from 37.8 dB to 80.9 dB (mean  = 64.9, SE = 0.9). Peak frequency ranged from 172 to 4307 Hz, falling within the hearing range of ungulates [73], and averaged 958 Hz (SE = 41), consistent with the low frequency of traffic noise [74]. Of all human activities measured, the number of autos passing was most strongly correlated with average power measurements during scan samples (r = 0.37), further pointing to traffic as a dominant source of noise. Of 1013 ungulates scanned across all scan samples, 234 (23%) were engaged in responsive behavior (14% traveling, 7% vigilant, 2% fleeing, and 0.2% defensive).

When comparing our candidate models predicting ungulate responsiveness, there was some model selection uncertainty (Table 1) with substantial support for the top 3 models (out of 33) that fell within 2.0 ΔAIC_c_ [67]; these top models contained all acoustic and human activity predictors except peak frequency. Based on the magnitude and direction of parameter estimates, ungulates were more likely to respond when there were more pedestrians present and less likely to respond when there were high levels of traffic, with traffic having a greater effect than pedestrians (Table 2). The 95% confidence intervals around the parameter estimates for total vehicles passing and pedestrians did not overlap zero in the top model or from model averaging, further suggesting that they both influenced responsiveness. The parameter estimate for average power was relatively small, and its 95% confidence interval overlapped zero when model averaging but not when average power was the sole predictor, suggesting only a weak negative relationship between noise and responsiveness. The parameter estimates for autos stopped and peak frequency were also small, with confidence intervals overlapping zero both from model averaging and when they were the only predictors (Table 2). Comparing the importance weights of the acoustic and human activity variables confirmed that the number of vehicles passing and pedestrians were relatively more important predictors of ungulate responsiveness than average power, the numbers of autos stopped, and peak frequency (Table 2). Based on the magnitude and directions of parameter estimates for the covariates, ungulates were more likely to respond when herds were dispersed, were closer to the road, and were composed of pronghorn, with at least one confidence interval that did not overlap zero from the top model, model averaging, or the model with a single predictor (Table 2).

**Table 1:** AIC_c_ model selection results where acoustic and human activity variables were used to explain whether or not individuals were responsive during scan samples.

Model^a^	K^b^	ΔAIC_c_	Model weight (*w_i_*)
total vehicles passing, pedestrians	11	0.0	0.214
total vehicles passing, pedestrians, autos stopped	12	1.8	0.087
total vehicles passing, pedestrians, average power	12	2.0	0.079
total vehicles passing	10	2.2	0.071
total vehicles passing, pedestrians, peak frequency	12	2.4	0.065
total vehicles passing, autos stopped	11	3.0	0.048
pedestrians, average power	11	3.3	0.041
pedestrians	10	3.5	0.037
total vehicles passing, pedestrians, average power, autos stopped	13	3.8	0.032

Covariates (distance to road, distance to cover, dispersion, herd size, species, Julian date, time of day) and a random effect (Herd ID) were also included in each model.

a The top 9 models (out of 33) that fell within 4 AIC_c_ of the top model (holding 67% of the total model weight) are presented.

b Parameter count for the model (including intercept and variance).

**Table 2:** Relative variable importance weights (for acoustic and human activity variables) and parameter estimates with 95% confidence intervals (for all variables, including covariates) from models predicting ungulate responsiveness in our scan samples.

Variable	Relative importance weight	Estimate from top model (lower/upper CL)	Estimate from model averaging (lower/upper CL)	Estimate from model with one predictor (lower/upper CL)
**Acoustic or human activity predictor:**				
total vehicles passing	0.76	−0.23 (−0.41/−0.05)*	−0.15 (−0.20/−0.11)*	−0.16 (−0.33/0.004)
pedestrians	0.70	0.11 (0.01/0.21)*	0.09 (0.05/0.12)*	0.09 (−0.01/0.20)
average power	0.33		−0.01 (−0.03/0.02)	−0.03 (−0.06/−0.01)*
autos stopped	0.33		−0.01 (−0.04/0.02)	0.07 (−0.06/0.21)
peak frequency	0.24		0 (−0.0001/0.0001)	0.0002 (−0.001/0.001)
**Covariate:**				
distance to road		−0.01 (−0.003/0.001)	−0.001 (−0.002/0.002)	−0.002 (−0.004/−0.0003)*
distance to cover		−0.001 (−0.33/0.004)	−0.001 (−0.004/0.002)	−0.30 (−1.38/0.78)
dispersion		1.34 (0.62/2.07)*	1.19 (0.44/1.93)*	1.08 (0.37/1.79)*
herd size		0.02 (−0.01/0.05)	0.01 (−0.03/0.05)	−0.01 (−0.03/0.01)
species		−1.02 (−1.78/−0.26)* a	−0.92 (−2.18/0.96)	−0.60 (−1.29/0.09)
Julian date		0.002 (−0.01/0.01)	0.002 (−0.01/0.01)	0.001 (−0.01/0.01)
time of day		0.56 (−0.22/1.34)	0.53 (−0.73/1.78)	0.58 (−0.18/1.34)

Parameter estimates and confidence intervals are presented for variables in the top model, for all variables based on model averaging across all 33 models, and from models containing each variable as a sole predictor of ungulate responsiveness.

* Confidence interval not overlapping zero.

a Indicates greater responsiveness of pronghorn than elk.

### Focal Samples

We conducted 113 focal samples throughout the field season generating 1,632 minutes of individual observations. We observed 3,275 autos stopped, 3,040 vehicles passing (including 2,786 autos, 171 trucks, 83 motorcycles), 1,047 pedestrians, and 41 bicycles passing summed over 2,172 human activity scans that were concurrent with the 113 focal observations. Our uncalibrated measures of average power during focal samples ranged between 57.0 dB and 77.0 dB (mean  = 69.2, SE = 0.4), while peak frequency ranged between 172 and 11,887 Hz (mean  = 958, SE = 74.1). Of the human activities measured, the number of autos passing was most strongly correlated with average power during focal samples (r = 0.54), again implicating auto traffic as a major source of noise. On average, focal ungulates spent 25% (SE = 2%) of their time engaged in responsive behavior (13% traveling, 8% vigilant, 4% fleeing, 0.1% defensive).

When comparing our candidate models predicting ungulate responsiveness, there was considerable model selection uncertainty (Table 3), with substantial support for the top 8 models (out of 129) that fell within 2.0 ΔAIC_c_ [67]; these top models contained all acoustic and human activity predictors. Based on the magnitude and direction of parameter estimates in the most strongly supported models, focal animals increased their responsiveness with increasing motorcycle traffic and decreased their responsiveness with increasing auto traffic, with motorcycles having a larger effect size than autos (Table 4). The 95% confidence intervals around the parameter estimates for these two predictors did not overlap zero in the top model, further suggesting they influenced responsiveness. In contrast, the parameter estimates for the other acoustic and human activity variables in the top model (average power and pedestrians) were relatively small, with confidence intervals that overlapped zero (Table 4). All model-averaged parameter estimates of acoustic and human activity variables were smaller than those from the highest-ranking models, with confidence intervals overlapping zero, suggesting that they did not strongly influence responsiveness, though this could be attributed to averaging over many models with high uncertainty, which may reduce the ability to correctly estimate the effect of a single predictor [69], [70]. Comparing the relative importance weights of the acoustic and human activity predictors revealed that the number of autos passing was the most important predictor of ungulate responsiveness followed by the number of motorcycles passing. Average power, pedestrians, autos stopped, peak frequency, and bicycles passing were relatively less important (Table 4). Further, based on the magnitude and directions of parameter estimates for the covariates (with at least one confidence interval that did not overlap zero from model averaging or the single-predictor model), ungulates were more responsive in smaller herds and during daytime hours, and cows with a calf were more responsive than males or females without a calf (Table 4).

**Table 3:** AIC_c_ model selection results where acoustic and human activity variables were used to explain the proportion of time individual focal animals were responsive.

Model^a^	K^b^	ΔAIC_c_	Model weight (*w_i_*)
autos passing, motorcycles passing, average power, pedestrians	15	0.0	0.070
autos passing, motorcycles passing, average power	14	0.0	0.069
autos passing, motorcycles passing, pedestrians	14	0.6	0.053
autos passing, motorcycles passing	13	0.7	0.049
autos passing, motorcycles passing, average power, autos stopped	15	1.4	0.034
autos passing, motorcycles passing, autos stopped	14	1.6	0.031
autos passing, motorcycles passing, average power, bicycles passing	15	1.8	0.028
autos passing, motorcycles passing, average power, pedestrians, peak frequency	16	2.0	0.026
autos passing, motorcycles passing, average power, pedestrians, bicycles passing	16	2.1	0.024
autos passing, average power	13	2.3	0.022
autos passing	12	2.3	0.022
autos passing, motorcycles passing, pedestrians, peak frequency	15	2.4	0.021
autos passing, pedestrians	13	2.6	0.019
autos passing, average power, pedestrians	14	2.7	0.018
autos passing, motorcycles passing, average power, peak frequency	15	2.7	0.018
autos passing, motorcycles passing, average power, pedestrians, autos stopped	16	2.7	0.018
motorcycles passing, autos stopped	13	2.8	0.018
autos passing, motorcycles passing, bicycles passing	14	2.9	0.016
autos stopped	12	3.0	0.016
autos passing, motorcycles passing, pedestrians, bicycles passing	15	3.0	0.015
autos passing, motorcycle passing, pedestrians, autos stopped	15	3.2	0.014
pedestrians	12	3.2	0.014
motorcycles passing, pedestrians	13	3.3	0.014
autos passing, motorcycles passing, peak frequency	14	3.3	0.014
autos passing, autos stopped	13	3.4	0.013
autos passing, motorcycles passing, average power, autos stopped, bicycles passing	16	3.5	0.012
autos passing, average power, autos stopped	14	3.8	0.011
autos passing, motorcycles passing, average power, autos stopped, peak frequency	16	3.8	0.010
autos passing, motorcycles passing, autos stopped, peak frequency	15	3.8	0.010

Covariates (distance to road, distance to cover, dispersion, herd size, species, Julian date, time of day, and sex) were also included in each model.

a The top 29 models (out of 129) that fell within 4 AIC_c_ of the top model (holding 70% of the total model weight) are presented.

b Parameter count for the model (including intercept and variance).

**Table 4:** Relative variable importance weights (for acoustic and human activity variables) and parameter estimates with 95% confidence intervals (for all variables, including covariates) from models predicting ungulate responsiveness in our focal observations.

Variable	Relative importance weight	Estimate from top model (lower/upper CL)	Estimate from model averaging (lower/upper CL)	Estimate from model with one predictor (lower/upper CL)
**Acoustic or human activity predictor:**				
autos passing	0.80	−0.08 (−0.14/−0.02)*	−0.05 (−0.19/0.08)	−0.06 (−0.11/−0.01)*
motorcycles passing	0.69	0.57 (0.06/1.09)*	0.37 (−0.09/0.82)	0.35 (−0.15/0.85)
average power	0.50	0.01 (−0.002/0.03)	0.005 (−0.01/0.02)	−0.0001 (−0.01/0.01)
pedestrians	0.46	−0.04 (−0.09/0.01)	−0.02 (−0.09/0.05)	−0.04 (−0.10/0.01)
autos stopped	0.34		−0.01 (−0.06/0.04)	−0.04 (−0.08/−0.002)*
peak frequency	0.25		−0.0003 (−0.01/0.01)	0 (−0.0001/0.01)
bicycles passing	0.24		0.04 (−0.44/0.53)	−0.48 (−1.37/0.42)
**Covariate:**				
distance to road		−0.0001 (−0.001/0.0004)	−0.0001 (−0.001/0.001)	−0.0001 (−0.001/0.0003)
distance to cover		0.34 (−0.09/0.77)	0.35 (−0.54/1.25)	0.13 (−0.41/0.68)
dispersion		0.03 (−0.07/0.14)	0.03 (−0.09/0.15)	0.04 (−0.07/0.15)
herd size		−0.005 (−0.01/0.0004)	−0.11 (−0.12/−0.10)*	−0.006 (−0.01/−0.0004)*
species		0.09 (−0.05/0.23)	0.08 (−0.14/0.30)	−0.06 (−1.04/0.92)
Julian date		−0.0002 (−0.002/0.001)	−0.0004 (−0.002/0.002)	−0.0005 (−0.002/0.001)
time of day		0.11 (−0.002/0.22)	0.09 (−0.15/0.33)	0.11 (0.01/0.22)* a
sex		−0.12 (−0.24/0.01)	−0.11 (−0.36/0.14)	−0.16 (−0.27/−0.04)* b

Parameter estimates and confidence intervals are presented for variables in the top model, for all variables based on model averaging across all 129 models, and from models containing each variable as a sole predictor of ungulate responsiveness.

* Confidence interval not overlapping zero.

a Indicates greater responsiveness during daytime hours than crepuscular hours.

b Indicates greater responsiveness of females with calf than males or females without a calf.

## Discussion

The risk-disturbance hypothesis states that anthropogenic disturbance such as human-related presence, objects, or sounds will elicit antipredator behavior [16]. Thus, we expected ungulates to exhibit heightened levels of responsive behavior in the presence of human activities and noise along Teton Park Road in Grand Teton National Park. The results suggest that human activities can alter responsive behaviors in ungulates. Contrary to our predictions, however, ungulates were not more likely to respond, but rather less likely to respond to increased vehicle traffic, which was the human activity most closely associated with noise. Though noise levels themselves did not have a strong effect on ungulate behavior, there was a weak negative relationship between average power and responsiveness in our scan samples.

One possible explanation for these findings is that ungulates in our study area did not perceive traffic and its associated noise as a form of predation risk, perhaps because individuals sensitive to these stimuli have been displaced over time or because the individuals that remain have habituated over time to these frequent stimuli. Ungulates are known to habituate to regular exposure to noise [5], [31] and other non-lethal human activities [36] and to display individual variation within populations in their avoidance or tolerance of roads [62]. Elk in particular exhibit behavioral patterns that suggest habituation along roads and other areas disturbed by human activities [75]–[77]. This tolerance would explain a lack of effect of traffic on responsiveness, but does not seem sufficient to explain the finding that increasing traffic caused ungulates to be *less* responsive.

The decrease in responsiveness with increasing traffic could indicate that passing vehicles provide a refuge from predators, such that ungulates have come to perceive reduced predation risk when traffic and their associated noise levels are high. Previous studies have demonstrated direct benefits of human activity to prey through reduced predator abundance [14], [32]–[35], and it is possible that this could also translate to indirect benefits through reduced investment in vigilance and other forms of antipredator behavior. Alternatively, another explanation for our findings is that traffic disturbances are actually perceived as a form of predation risk by ungulates, but they cannot afford to maintain high levels of responsiveness to such a continuous and pervasive form of disturbance. Specifically, the risk allocation hypothesis [78] suggests that animals will devote a larger proportion of risky intervals to antipredator behavior, when those intervals are brief and infrequent. In contrast, when periods of risk are lengthy and more frequent, animals will devote a reduced proportion of those risky intervals to antipredator behavior in order to avoid the high cost of lost foraging. In the context of anthropogenic disturbance, Miller et al. [79] found certain human activities, when infrequent and unpredictable, were related to heightened levels of flush distance in ungulates. In our study, auto traffic, with its associated noise, was the most prevalent anthropogenic disturbance; thus, high traffic levels may have reduced responsiveness due to risk allocation decisions. In comparison, pedestrians, a less frequent form of disturbance, were more likely to elicit responsive behavior in our scan samples, consistent with prior studies implicating the human form as an importance source of disturbance for ungulates [36]. Similarly, responsiveness was greater in response to the least common form of disturbance, motorcycle traffic, as would be predicted by the risk allocation hypothesis. Interestingly, bicycles, which are quieter but similar in shape to motorcycles, were not an important predictor of responsive behavior, suggesting that the loud noise generated by motorcycles in particular may be a disturbance stimulus.

Although the goal of this study was to evaluate whether anthropogenic disturbances affected ungulate behavior, we also measured a variety of covariates for inclusion in our models. The directions of their effects on responsiveness supports earlier findings that ungulates were more responsive when they were in smaller herds, when they were dispersed rather than clustered, and when they were closer to roads, further suggesting they were not completely tolerant of human activity [36], [39], [58], [66]. Our results also suggest that ungulates may be more responsive during daytime hours; this adds to prior findings that time of day influences responsiveness, though the direction of the effect varies across ungulate species and populations, including elk [36], [64]. Pronghorn were more responsive than elk, and females with young were more responsive than adult males and adult females without young, again consistent with prior studies demonstrating the sensitivity of pronghorn [39], [71], [80] and of females with young [21], [71], [81] to disturbance.

Understanding the behavioral responses of wildlife to anthropogenic disturbance can have important conservation and management implications [82]–[86]. Our results highlight an interesting effect of human disturbance on behavior. Except in the case of motorcycles, which are relatively infrequent disturbance events, ungulates spent less time responding with increased vehicle traffic and its associated noise, allowing more time for maintenance activities such as feeding. Presumably, increased levels of energy enhancing activities can positively affect fitness, suggesting a benefit of reduced responsiveness to traffic. However, we urge caution with this interpretation, since unresponsive behavior also could have negative implications, for example by reducing their ability to visually detect predators and other cues in the environment, potentially adding to any masking of acoustic cues caused by the anthropogenic noise itself [1]. Reduced responsiveness of ungulates to road traffic could also lead to increased human conflict such as negative encounters with recreationists or collisions with vehicles [33], [87], major concerns for park managers [88]. Finally, it is important to emphasize that noise can have negative impacts on fitness and population persistence in ways that may not be reflected by individual behavioral responsiveness [89]. Thus, although anthropogenic noise did not appear to detract from fitness-enhancing behaviors in this system, we suggest continued investigation of possible population-level noise impacts.
